# Insight Into Genomic Changes Accompanying Divergence: Genetic Linkage Maps and Synteny of *Lucania goodei* and *L. parva* Reveal a Robertsonian Fusion

**DOI:** 10.1534/g3.114.012096

**Published:** 2014-06-03

**Authors:** Emma L. Berdan, Genevieve M. Kozak, Ray Ming, A. Lane Rayburn, Ryan Kiehart, Rebecca C. Fuller

**Affiliations:** * Department of Animal Biology, University of Illinois, Champaign, Illinois 61820; † Department of Plant Biology, University of Illinois, Urbana, Illinois 61801; ‡ Department of Crop Sciences, University of Illinois, Urbana, Illinois 61801; § Department of Biology, Ursinus College, Collegeville, Pennsylvania 19426

**Keywords:** synteny, Robertsonian fusion, chromosomal rearrangement, linkage map, speciation, EST-based SNPs, fundulidae

## Abstract

Linkage maps are important tools in evolutionary genetics and in studies of speciation. We performed a karyotyping study and constructed high-density linkage maps for two closely related killifish species, *Lucania parva* and *L. goodei*, that differ in salinity tolerance and still hybridize in their contact zone in Florida. Using SNPs from orthologous EST contigs, we compared synteny between the two species to determine how genomic architecture has shifted with divergence. Karyotyping revealed that *L. goodei* possesses 24 acrocentric chromosomes (1N) whereas *L. parva* possesses 23 chromosomes (1N), one of which is a large metacentric chromosome. Likewise, high-density single-nucleotide polymorphism−based linkage maps indicated 24 linkage groups for *L. goodei* and 23 linkage groups for *L. parva*. Synteny mapping revealed two linkage groups in *L. goodei* that were highly syntenic with the largest linkage group in *L. parva*. Together, this evidence points to the largest linkage group in *L. parva* being the result of a chromosomal fusion. We further compared synteny between *Lucania* with the genome of a more distant teleost relative medaka (*Oryzias latipes*) and found good conservation of synteny at the chromosomal level. Each *Lucania* LG had a single best match with each medaka chromosome. These results provide the groundwork for future studies on the genetic architecture of reproductive isolation and salinity tolerance in *Lucania* and other Fundulidae.

Species-specific linkage maps are critical to understanding genomic architecture and how it differs between species. Linkage maps allow the exploration of genotype-phenotype relationships through quantitative trait loci mapping (Falconer and Mackay 1996; for examples, see Peichel *et al.* 2001; Tripathi *et al.* 2009) and the comparison of genomic architecture between species via synteny mapping (Prince *et al.* 1993; Backstrom *et al.* 2008; Muchero *et al.* 2009; Lucas *et al.* 2011; McGraw *et al.* 2011). However, the majority of previous studies of genome-wide synteny have used pairs of species that are distantly related (*i.e.*, from different orders or families: Stapley *et al.* 2008; Jaari *et al.* 2009; Foley *et al.* 2011; McGraw *et al.* 2011). It is less common for comparisons of synteny to be made within the same genus (but see Ming *et al.* 1998; Rogers *et al.* 2007; Lubieniecki *et al.* 2010; Lee *et al.* 2011; Timusk *et al.* 2011; Naish *et al.* 2013). High-density linkage maps are now possible with the advent of high-throughput sequencing and have the potential to facilitate fine-scale comparisons of synteny between closely related species. These comparisons are needed to determine how genome structure diverges and potentially contributes to speciation.

The problem for maintaining species boundaries in areas of sympatry is that gene flow between species and recombination in hybrids should homogenize species-specific traits and break down reproductive isolating barriers. Genomic rearrangements, such as chromosomal fusions, inversions, or deletions, can potentially facilitate the maintenance of reproductive isolating barriers because they reduce recombination in portions of the genome (Rieseberg 2001; Butlin 2005; Faria and Navarro 2010). Data from a wide range of taxa support the theory that genes conferring reproductive isolation between sympatric species often are localized to rearranged areas of the genome (Rieseberg *et al.* 1999; Noor *et al.* 2001; Coluzzi *et al.* 2002; Feder *et al.* 2003; Kitano *et al.* 2009).

One way to study the role of genomic rearrangements in speciation is to compare synteny between species that hybridize at low levels. We do this in *Lucania*, a genus that is becoming a model system for ecological speciation research. The bluefin killifish (*Lucania goodei)* and the rainwater killifish (*L. parva*) are two closely related species that differ radically in their salinity tolerance (Duggins *et al.* 1983; Whitehead 2010). *Lucania goodei* is found primarily in freshwater habitats, whereas *L. parva* is euryhaline and can be found in fresh, brackish, and marine habitats (Lee *et al.* 1980). Survival at various life stages differs between *Lucania* species in different salinities (Fuller *et al.* 2007; Fuller 2008). *Lucania goodei* and *L. parva* also show divergence in sequence and expression of a number of salinity tolerance genes (Berdan and Fuller 2012; Kozak *et al.* 2014). Despite these ecological differences between species, there is evidence for low levels of ongoing hybridization in sympatric populations. Sympatric populations exist in the Atlantic and Gulf coastal waterways of Florida (Fuller and Noa 2008). Hubbs *et al.* (1943) found hybrids based on morphological characters in one population, and analysis of mtDNA suggests recent gene flow between the two species in multiple river drainages in Florida (R. C. Fuller, unpublished data). We hypothesized that differences in genome structure might contribute to speciation in *Lucania*. This hypothesis was sparked by an unsupported comment made in an older study; Uyeno and Miller (1971) stated that chromosome number differs between *L. goodei* and *L. parva* but did not provide any evidence to support this statement.

The purpose of this study was to determine whether *L. goodei* and *L. parva* differ in genomic architecture. To do this, we (1) karyotyped both species and (2) produced two high-density single-nucleotide polymorphism (SNP)-based linkage maps. Transcriptome sequencing and high-throughput SNP genotyping were performed in both species to create the linkage maps. These data allowed us to determine (1) the number of chromosomes possessed by each species, (2) whether a fusion/fission event had occurred, and (3) patterns of synteny between the two species. We analyzed synteny at the linkage group (LG) level and at the level of marker order to determine whether large or small-scale genomic rearrangements have occurred during divergence (Gale and Devos 1998; Ming *et al.* 1998; Wang *et al.* 2010). We further compared synteny in *Lucania* with the most closely related species with a sequenced genome, medaka (*Oryzias latipes*) to ask how *Lucania* genomic architecture corresponds to that of other teleost fish (Kasahara *et al.* 2007). These linkage maps will enable future studies to ask whether the areas of the genome contributing to salinity tolerance also are implicated in reproductive isolation and whether these traits map to genomic rearrangements in *Lucania*.

## Materials and Methods

### Karyotyping

Somatic karyotypes were determined for both *L. goodei* and *L. parva* using metaphase spreads. For each species, animals of both sexes from multiple populations were used. Details on the karyotyping methods can be found in Supporting Information, File S1. In summary, individuals were injected intraperitoneally at 0 hr with a phytohemagglutinin solution to stimulate mitosis, injected at 24 hr with 1% colchicine solution, and then killed at 26 hr using MS-222. The gills were removed, placed in chilled distilled water to allow the cells to swell (30 min), then fixed in a 3:1 methanol/glacial acetic acid mixture (30 min). The gills were dabbed on the slides to break the cells and release the chromosomes. Slides were cleared, dried, stained with 5% Giemsa solution (Electron Microscopy Sciences, Hatfield, PA), mounted with Permount, and visualized using a compound microscope.

### Comparative genetic linkage maps: overview

**Figure 1:**
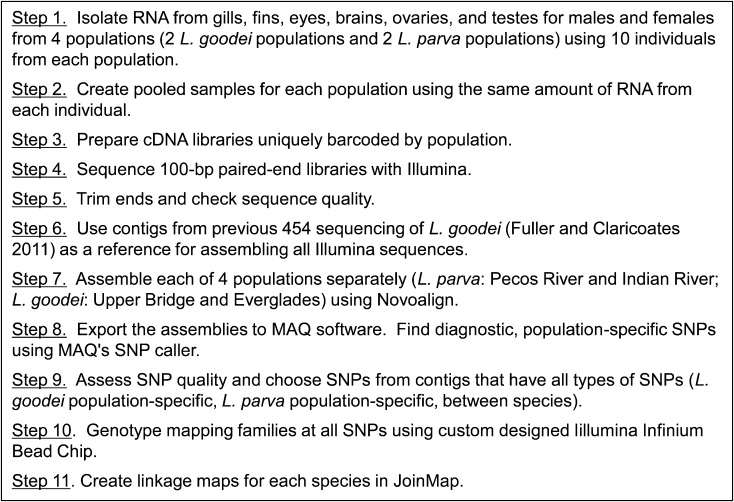
Procedures used to generate single-nucleotide polymorphisms (SNPs) from orthologous expressed sequence tags (ESTs) for linkage maps.

Genetic linkage maps were created separately for both *L. goodei* and *L. parva* from F_2_ mapping crosses between two geographically isolated populations. The key to comparing the genetic linkage maps between the two species was to use SNP markers from orthologous expressed sequence tags (ESTs; from 454 and Illumina sequencing) that were expressed in both species (Figure 1). F_2_ offspring and F_1_ parents were genotyped using an Illumina Infinium Bead Chip custom designed for *Lucania*.

### Mapping crosses

For each species, crosses were set up between geographically and ecologically divergent populations. For *Lucania goodei*, these populations were Upper Bridge from the spring-fed Wakulla River (Wakulla County, Florida) and a swamp population in the Everglades (Broward County, Florida). For *Lucania parva*, the populations were Indian River Lagoon, an Atlantic coast population where salinity is typically 35 ppt (Brevard County, Florida) and Pecos River, a freshwater inland river in Texas (Pecos-Crockett County border, Texas). Between population breeding pairs were established in both hybrid cross directions. Cross designs for the creation of the F_1_ parents are described for *L. goodei* in Fuller *et al.* (2010) and for *L. parva* in Kozak *et al.* (2012). F_1_ hybrid offspring were then raised to adulthood and paired with unrelated F_1_ hybrid individuals to create F_2_ offspring. For *L. parva*, we created and genotyped 161 F_2_ offspring from 8 F_2_ families and 16 F_1_ parents (parents from 11 F1 families: 4 Indian River female × Pecos male crosses; 7 Pecos female × Indian River male crosses; see Kozak *et al.* 2012). For *L. goodei*, we created and genotyped 303 F_2_ offspring from 14 F_2_ families and 28 F_1_ parents (21 F_1_ families: 11 Upper Bridge female × Everglades male crosses; 10 Everglades female × Upper Bridge male crosses).

F_2_ eggs were collected and raised in freshwater with dilute methylene blue (an antifungicide). Fry were fed newly hatched *Artemia* and raised to 1 month postfertilization. The fry were then killed in MS-222, preserved in ethanol, and stored at −80°. F_1_ parents and F_0_ grandparents were preserved in ethanol. F_2_ family sizes ranged from 15 to 24 fry.

### Creation of population-specific EST libraries

RNA was extracted from five males and five females from the two *L. goodei* populations (Upper Bridge and Everglades) and the two *L. parva* populations (Indian River and Pecos River). Fish were killed in MS-222. Tissue samples were taken from the gills (1−2 arches), dorsal fins, eyes, brain, and the gonads (ovaries or testes). RNA was extracted using a protocol modified from Carleton (2011: see File S1). A pooled sample containing RNA from all tissues was created for each population that contained equal amounts of RNA from all individuals. The RNA was submitted to the Keck Center for Comparative and Functional Genomics at the University of Illinois for creation of cDNA libraries and sequenced using Illumina HiSequation 2000 (see File S1). The two populations from *L. goodei* were uniquely barcoded and run on a single lane of Illumina HiSeq for 100-bp paired-end sequencing. Indian River *L. parva* and Pecos River *L. parva* were run on separate lanes. Average quality scores were above 20 for all cycles. Samples produced from 5 to 10 billion bases of data (reads per end: Upper Bridge = 29,661,140, Everglades = 26,235,855, Pecos River = 55,535,778, Indian River = 53,061,233). The population specific Illumina transcriptome sequences are archived in Genbank (bio-project ID: PRJNA215087).

In addition, 454 sequencing was done on pooled samples of RNA from five *L. goodei* populations across Florida (1: Upper Bridge Wakulla River, Wakulla, Co., FL; 2: St. Mark’s National Wildlife Refuge Gambo Bayou, Wakulla, Co., FL; 3: 26-Mile Bend, Everglades, Broward Co., FL; 4: Rum Island Park, Santa Fe River, Columbia Co., FL; and 5: Delk’s Bluff Bridge, Oklawaha River, Marion Co., FL). These sequences were used to construct a reference upon which subsequent assembly was based (see section: *Assembly and alignment*; reference available in Dryad accompanying this paper). Tissue samples were taken from the gills, fins, eyes, brain, and gonads (ovaries or testes). Additional details on the 454 project can be found in Fuller and Claricoates (2011).

### Assembly and alignment

*Lucania goodei* 454 sequences were used as a reference for the assembly. For the reference, contigs were assembled using Newbler assembler (454 Life Sciences, Branford, CT). Assembly parameters were as follows: the minimum contig length was set at 200 bp, the minimum overlap length was 60 bp, and the minimum overlap identity was 95%. A total of 29,838 contigs were generated by the Newbler assembler. Using the *L. goodei* 454 contigs as a reference anchored the analysis of shorter Illumina sequences and allowed identification of contigs that contained multiple SNPs. The goal was to find contigs containing a SNP that was diagnostic for the two *L. goodei* populations as well as a SNP that was diagnostic for the two *L. parva* populations. This method may have missed *L. parva* contigs that were not expressed in *L. goodei*, but these contigs are uninformative for the comparison of linkage maps. Illumina sequences were trimmed to 75 bp in length and aligned against the reference using Novoalign (Novocraft Technologies; www.novocraft.com). Alignment parameters were as follows: the maximum alignment score acceptable for a best alignment was set at 45; the gap extend penalty was set at 10; and the number of good quality bases for an acceptable read was set at 50.

### SNP selection

The alignments were exported to MAQ (*i.e.*, Mapping and Assembling with Quality) software (see Fuller and Claricoates 2011 for details) for SNP detection. Diagnostic SNPs for each population were identified using its population pair as a reference. SNPs were considered to be diagnostic when they were identified unambiguously for both populations. There were many more diagnostic SNPs between the two *L. parva* populations than there were between two *L. goodei* populations. To increase the number of SNPs for *L. goodei*, SNPs were used that were fixed in one population but were segregating in the alternate population.

Candidate SNPs were submitted to Illumina for initial evaluation for suitability for the Infinium Genotyping Assay. There were three classes of SNPs: *L. parva*−specific SNPs were SNPs that were segregating within *L. parva*, *L. goodei*−specific SNPs were SNPs that were segregating within *L. goodei*, and between species SNPs that were fixed (or nearly fixed) between the two species. The between species SNPs were designed for another study. The custom Infinium bead chip held probes for 4545 SNPs. Of these, 1497 were candidate SNPs for *L. goodei*, 1369 were candidate SNPs for *L. parva*, and 1679 were candidate between species SNPs. All SNPs were labeled with the ID number of the contig in which they occur. Protein annotations for the linkage map contigs were obtained using blastX searches against teleost reference proteomes (Atlantic killifish: *Fundulus heteroclitus*, Japanese medaka: *Oryzia latipes*, three-spined stickleback: *Gasterosteus aculeatus*, guppy: *Poecilia reticulata*) and human complete proteome (Uniprot release 2012_8 available at: ftp://ftp.uniprot.org/pub/databases/uniprot/previous_releases/release-2012_08/uniref/). Only contigs that matched a single protein in each species with a blast score >100 were considered high confidence annotations (Table S1). Proteins that matched multiple contigs located on different LGs were removed from the annotations.

### DNA extraction and SNP genotyping

DNA was extracted using a modified version of the PureGene (Gentra Systems; www.gentra.com) extraction protocol over 4 days (see File S1). Sample concentration and quality were verified using a Nanodrop spectrophotometer (Thermo Fisher Scientific, Waltham, MA). DNA samples were diluted to 75 µg/µL in nuclease-free water and then genotyped at all SNPs using the *Lucania* Illumina Infinium Bead Chip. Source and probe sequences from the chip are accessible on Dryad (http://datadryad.org/resource/doi:10.5061/dryad.hv75h/9). Bead chips were scanned using the iScan System (Illumina) at the Keck Center for Comparative and Functional Genomics at The University of Illinois. Illumina GenomeStudio (v2011.1) was used for genotype calls. Cluster positioning was performed separately for *L. goodei* and *L. parva* SNPs (no-call threshold was set to 0.15). Population-specific SNP alleles were verified from genotypes of 16 Upper Bridge *L. goodei* (7 males, 9 females), 17 Everglades *L. goodei* (7 males, 10 females), 6 Indian River *L. parva* (3 males, 3 females), and 5 Pecos River *L. parva* (3 males, 2 females). Genotype data were analyzed separately for each family. For each family, SNPs were removed if either (1) genotypes were homozygous in both parents thereby guaranteeing all offspring were homozygotes, or (2) the genotype was a no-call (*i.e.*, the sample did not run) for one or both parents. Not all SNPs were fixed between the two *L. goodei* populations, so we also genotyped the F_0_ grandparents to clarify phases.

### Linkage map creation

For each species, individual maps were constructed for each individual F_2_ family (14 for *L. goodei*; 8 for *L. parva*) using JoinMap 4.1 (Li *et al.* 2008). Parental and grandparental genotypes provided phase information for each locus. Grouping thresholds of LOD 4.0 (*L. goodei)* and 3.5 (*L. parva*) were used and markers significantly out of Hardy-Weinberg equilibrium (*P* < 0.001) were excluded. The Kosambi mapping function (Kosambi 1944) was used to convert recombination frequencies to cM. For each species, a consensus map was constructed using the Map Integration tool in JoinMap from the individual family map inputs. Linkage groups from individual families were joined if they shared two or more markers. MapChart 2.2 (Stam 1993) was used for graphical representation of the consensus map for each species. *Lucania parva* LGs were numbered in descending order based on total length in cM. *Lucania goodei* LGs were numbered based upon synteny with *L. parva* LGs (see section: *Synteny comparisons*).

### Synteny comparisons

To compare synteny between *L. parva* and *L. goodei*, SNPs designed from a common contig were considered to be putatively orthologous. Consistent clusters of SNPs from common contigs in both species provided evidence of synteny at the LG level (*i.e.*, SNPs from the same contigs clustered together in both species). Within LGs, marker order was compared among orthologous SNPs by performing rank order correlations between species. *Lucania goodei* and *L. parva* LGs also were compared with chromosomes from medaka (*Oryzias latipes*), the most closely related species with a fully sequenced genome [both are in the superorder Acanthopterygii (Steinke *et al.* 2006)]. Sequences of contigs in the *Lucania* linkage maps were blasted against medaka sequences using blastn. If a given contig had highly significant blast hits (bit score > 100) against a single medaka LG, then its approximate position was estimated in the medaka genome. However, if a given contig had multiple, highly significant blast hits on multiple medaka LGs, then the orthologous location in the medaka genome could not be determined. Hence, synteny was examined at the level of LG clustering for the *L. goodei* and *L. parva*, *L. goodei* and medaka, and *L. parva* and medaka comparisons. Synteny was examined at the level marker order for only the *L. goodei* and *L. parva* comparison. False-discovery rate *P*-values were calculated for rank order correlations using fdrtool in R (Strimmer 2008).

### Flow cytometry

The genome size of both species was estimated using flow cytometry to determine how much recombination was occurring per megabase. Four *L. goodei* and four *L. parva* individuals were collected from the Lower Bridge site on the Wakulla River, Florida. The DNA content of erythrocyte nuclei was measured using flow cytometry at Ursinus College (see File S1). *Betta splendens* was used as a standard.

## Results

**Figure 2:**
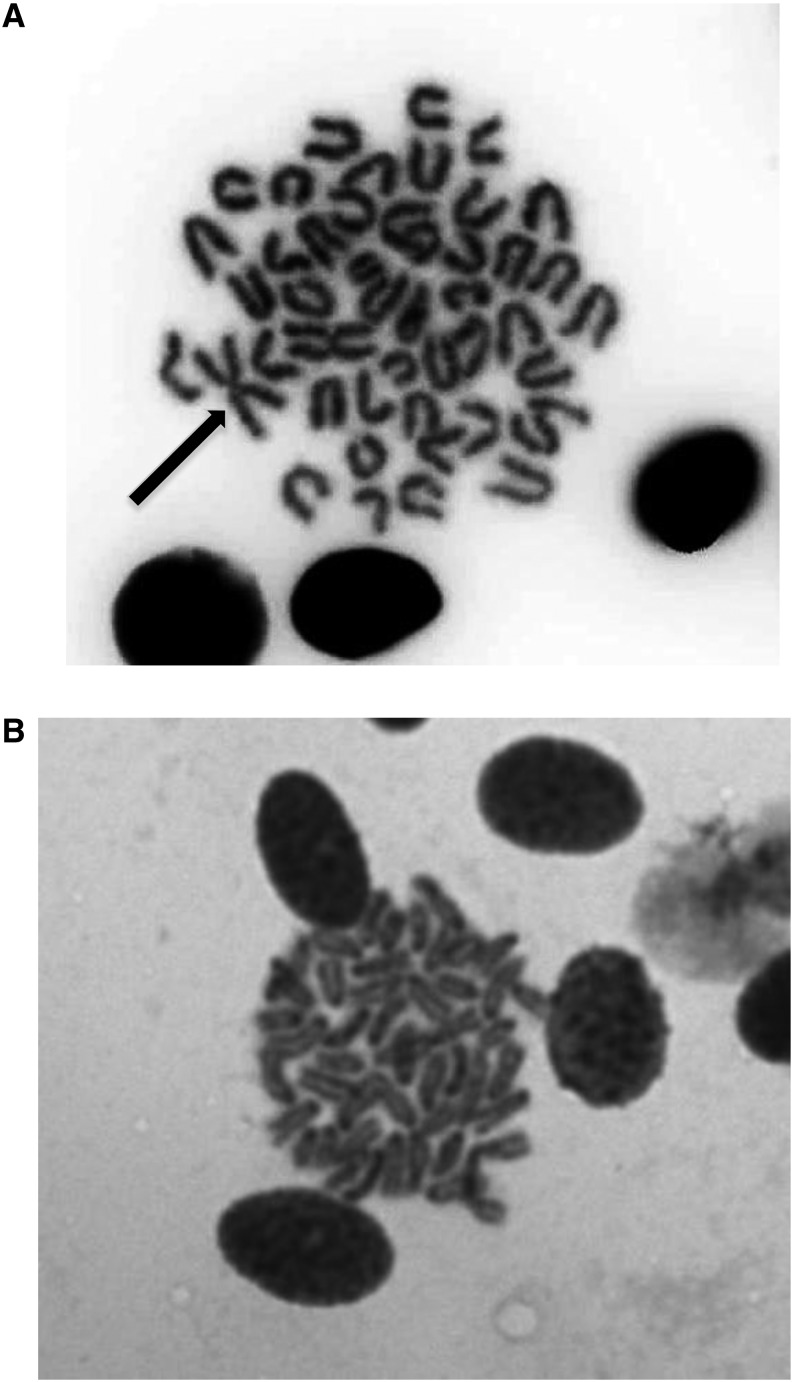
Somatic metaphase/anaphase spread of (A) *Lucania parva* and (B) *L. goodei*. The arrow in (A) indicates the fused chromosome.

The two *Lucania* species differed in chromosome number. *Lucania parva* had 23 chromosomes (Figure 2A). Twenty-two of these were acrocentric chromosomes of similar size, and one was a metacentric chromosome approximately twice the size of the other chromosomes (denoted by an arrow in Figure 2A). *Lucania goodei* had 24 acrocentric chromosomes (Figure 2B).

**Figure 3:**
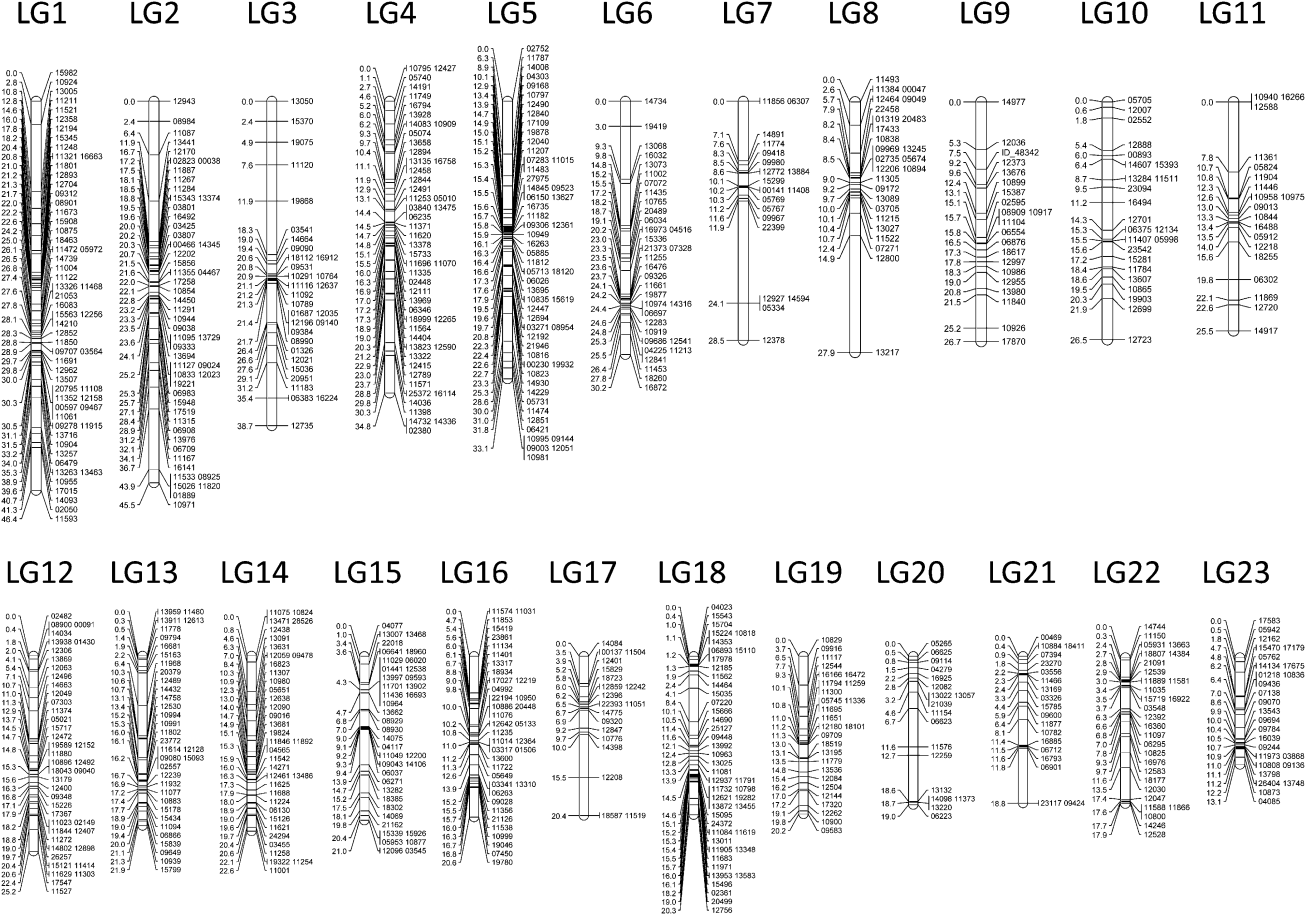
*Lucania parva* linkage map. Numbers on the right of each linkage group indicate the marker name and numbers on the left indicate the position in centimorgans (cM).

The number of chromosomes corresponded well to the number of LGs that were recovered. For *L. parva*, 23 linkage groups were found, which matched the number of chromosomes observed in the karyotype. Specifically, 766 SNP markers were resolved into 23 LGs (Table 1; Figure 3). Many of these SNP markers came from an EST that corresponded to a known protein, many of which could be assigned a putative function (Table S1). The number of markers per linkage group (LG) ranged from 18 (LG 20) to 59 (LG 1). The total length of the map was 605 cM with the average LG being 26.3 cM (Table 1). Marker density was 1.26 markers per cM on average spanning from 0.67 (LG 7) to 2.22 (LG 18). Marker density was relatively consistent with the largest gap being 13 cM on LG 8 and with 32 gaps of 4 cM or larger across the map. Average genome size (C-value) in *L. parva* was 1.423 pg (range: 1.396-1.450). One centimorgan in *L. parva* is thus approximately 2.3 Mb.

**Table 1:** Summary of integrated linkage maps for *L. parva* and *L. goodei*

	*L. parva*	*L. goodei*
No. chromosomes	23	24
No. linkage groups	23	24
Map size, cM	605	392
Average linkage group size, cM	26.3	16.3
Markers per cM	1.26	2.41
Total no. markers	766	915
No. individuals	161	303

**Figure 4:**
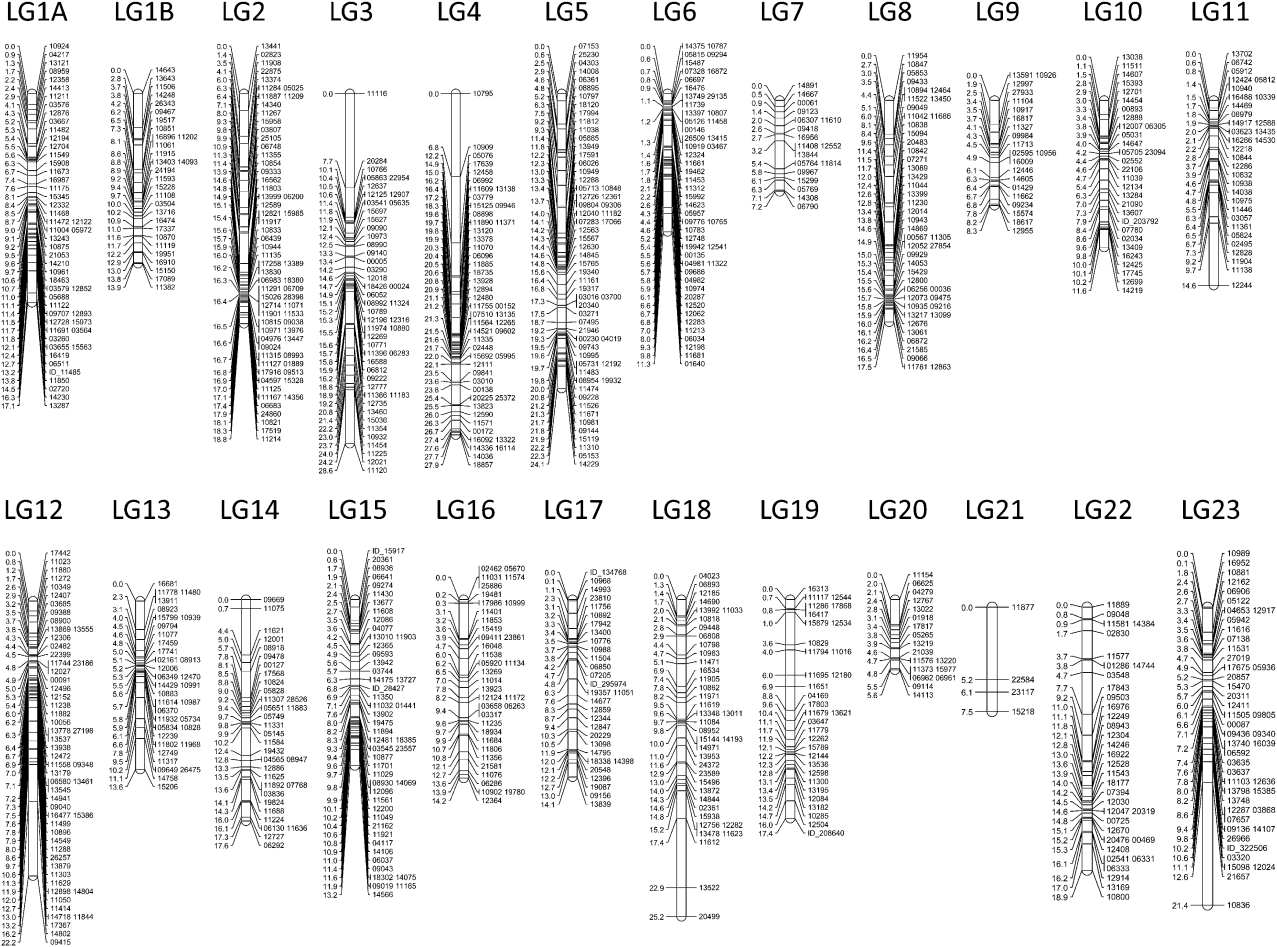
*Lucania goodei* linkage map. Numbers on the right of each linkage group indicate the marker name and numbers on the left indicate the position in centimorgans (cM).

Similarly, the number of LGs recovered for *L. goodei* matched the number of chromosomes (1N = 24). Significant linkages were found for 915 SNP markers making up 24 LGs (Table 1; Figure 4). Again, many of these SNP markers came from an EST that corresponded to a known protein (Table S1). The number of markers per LG ranged from 4 (LG 21) to 66 (LG 2). The linkage map spanned 392 cM with the average LG size being 16.33 cM (Table 1). Average marker density was 2.41 markers per cM, ranging from 0.53 (LG 21) to 4.42 (LG 6). Marker density was consistent with the largest gap being 8.7 cM on LG 23 and only 9 gaps of 4 cM or larger across the entire map. Average genome size in *L. goodei* was 1.349 pg (range: 1.342-1.356). One centimorgan in *L. goodei* is thus approximately 3.44 Mb.

### Synteny of *L. parva* and *L. goodei* maps

**Figure 5:**
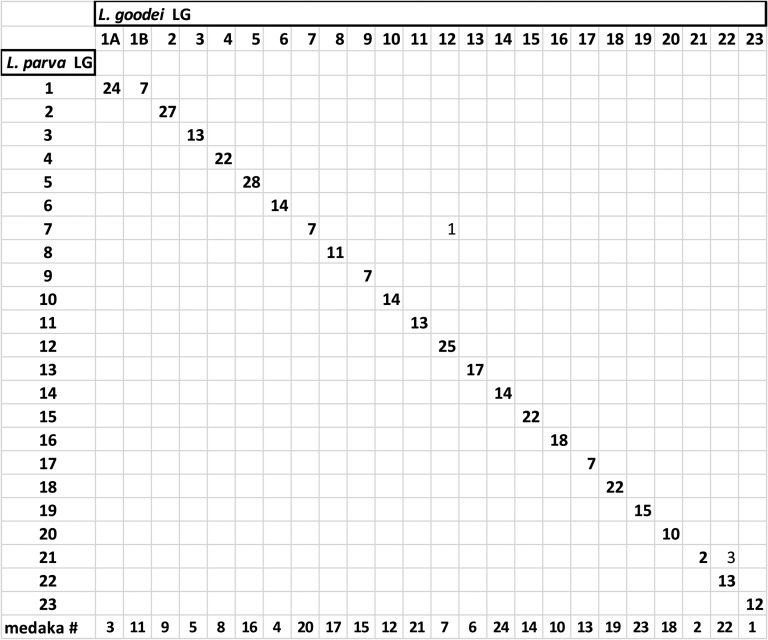
Summary of synteny comparisons between *L. parva* and *L. goodei* linkage groups. Bolded numbers along the diagonal show the number of orthologous single-nucleotide polymorphisms on the linkage groups. Numbers off the diagonal are nonsyntenic markers. Identity of syntenic medaka chromosomes listed along the bottom row.

The LGs were highly syntenic between *L. goodei* and *L. parva*, allowing us to unambiguously assign orthologous LGs. Across all LGs, we found 368 markers shared between the linkage maps that were from putatively orthologous ESTs. Figure 5 shows that markers from the putatively orthologous ESTs clustered together in the same LGs in *L. goodei* and *L. parva*. Specifically, 364 (98.91%) markers (those from a common contig and in both linkage maps) showed a pattern of synteny, whereas only 4 markers (1.09%) deviated and clustered differently in the two species (Figure 5 and Figure S1).

**Figure 6:**
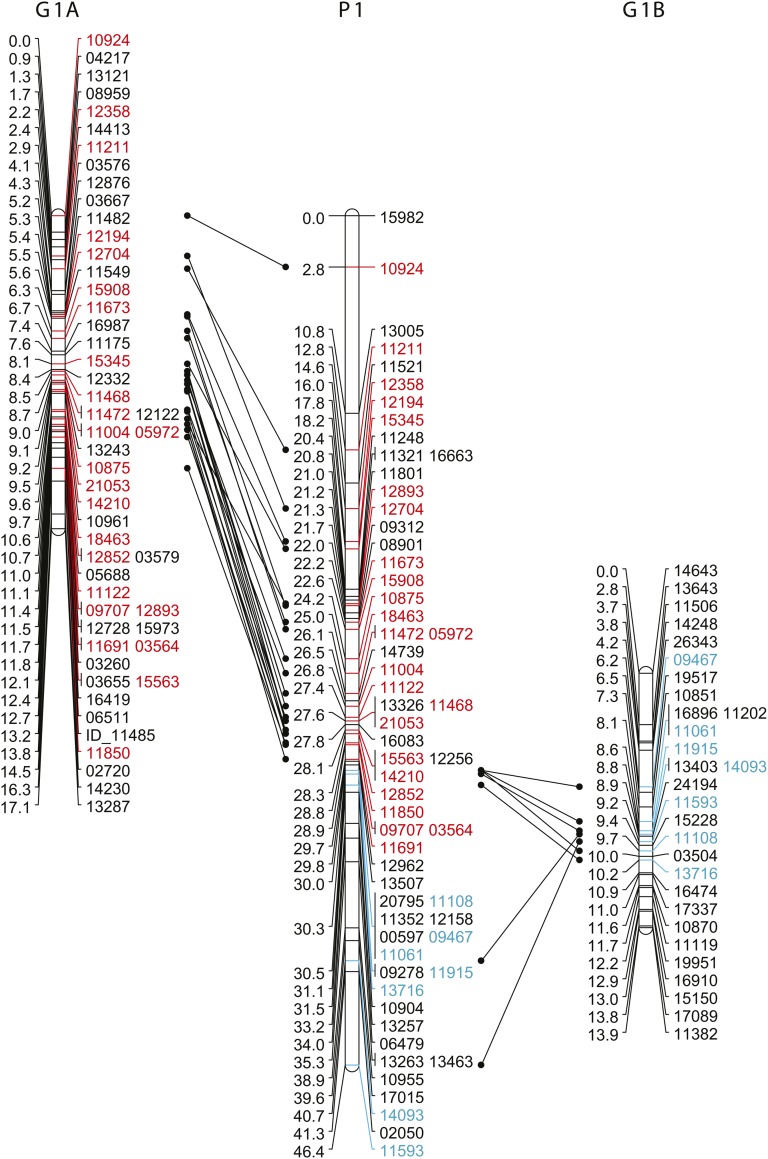
Synteny of linkage group 1 in *L. parva* and 1A,B in *L. goodei*. Orthologous single-nucleotide polymorphisms (SNPs) between *L. parva* linkage group 1 (P: middle) and *L. goodei* (G) linkage groups 1A (far left) and 1B (far right) are shown connected by lines. SNPs syntenic with *L. goodei* 1A are highlighted in red; SNPs syntenic with *L. goodei* 1B are highlighted in blue.

Two LGs from *L. goodei* were syntenic with the largest LG in *L. parva*, strongly suggesting that LG 1 represents the metacentric chromosome observed in the *L. parva* metaphase spread. Figure 6 shows that LG 1A in *L. goodei* was syntenic with the top portion of LG 1 in *L. parva* (matching at 24 markers: Figure 5), and LG 1B was syntenic with the bottom portion (matching at 7 markers).

Synteny was less conserved at the level of marker order within LGs (Table 2). Rank order correlations were high for some LGs but were low and nonsignificant for others. Across all LGs combined, marker order was highly correlated in *L. parva* and *L. goodei* (Spearman rank order correlation: n = 364, r = 0.99, *P* < 0.0001). When rank order correlations were performed on LGs separately (and corrected for multiple testing by use of the false-discovery rate), marker order was significantly correlated in 11 of 23 syntenic LGs (50%; Table 2; note that LG21 lacked sufficient orthologous markers to test marker order). The marker order correlations were not statistically significantly greater than zero for the other 12 LGs. To determine whether noncoding repetitive RNAs or slight differences in the order of densely mapped markers were influencing this result, we repeated these synteny analyses using only syntenic markers ≥0.5 cM apart in either species that had a single identified location in the medaka genome. Again, we found significant marker order preservation in less than half (38%) of the groups (6 of 16 with more than 5 syntenic markers).

**Table 2:** Marker order correlations for syntenic markers on each linkage group in *Lucania parva* and *L. goodei*

LG	Size *L. parva*, cM	Size *L. goodei*, cM	No. Syntenic Markers	Spearman r	*P* Value	FDR-Corrected *P* Value
1	46.4	A:17.1	24	0.8183	0.000001	0.000084
		B:13.9	7	0.607	0.1362	0.251906
2	45.5	22	27	0.835	0.000001	0.000084
3	38.7	28.6	13	0.4341	0.137904	0.251906
4	34.8	27.9	22	0.9198	0.000001	0.000084
5	33.5	26	28	0.7225	0.000014	0.007719
6	30.2	11.3	14	0.033	0.914228	1.000000
7	28.5	7.2	7	0.785714	0.0536	0.251906
8	27.9	17.5	11	0.6818	0.02071	0.030374
9	26.7	8.3	7	0.214286	0.5962	1.000000
10	26.5	11.6	14	0.3626	0.20193	1.000000
11	25.5	14.6	13	0.1923	0.529033	1.000000
12	25.2	22.2	25	0.5354	0.005817	0.030374
13	22.7	13.8	17	0.348	0.170412	0.251906
14	22.6	18.4	14	0.5956	0.02454	0.094289
15	21	13.2	21	0.441	0.039729	0.094289
16	20.6	15.3	18	0.5542	0.017116	0.030374
17	20.4	14.1	7	0.142857	0.7264	1.000000
18	20.3	30	22	0.8701	0.000001	0.000084
19	20.2	17.9	15	0.7214	0.002382	0.007719
20	19	9.5	10	0.3455	0.328748	1.000000
21	18.8	7.5	2	N/A	N/A	N/A
22	17.9	22.1	13	0.7637	0.002393	0.021931
23	13.1	21.4	12	0.6783	0.0153	0.030374

LG, linkage group; FDR, false-discovery rate.

### Synteny of *Lucania* and medaka

Synteny at the LG level was also well preserved between *Lucania* and medaka (Figure S2). Medaka has 24 chromosomes, and all *Lucania* LGs had a single best match to a medaka chromosome (listed at the bottom of Figure 5). Between *L. goodei* and medaka 559 of 585 (95.6%) orthologous markers were syntenic at the LG level. Similarly, *L. parva* had 532 of 545 (97.6%) orthologous markers that were found to be syntenic with medaka chromosomes. LG 1A in *L. goodei* corresponded to chromosome 3 in medaka and LG 1B corresponded to medaka chromosome 11.

## Discussion

Using high-throughput Illumina Infinium genotyping assays, we created two SNP-based linkage maps for *Lucania parva* and *L. goodei* with high marker density (1.26 markers/cM in *L. parva* and 2.41 markers/cM in *L. goodei*). These linkage maps establish genomic resources for *Lucania* and provide the groundwork for future linkage disequilibrium studies, quantitative trait loci mapping, molecular population genetic studies, and further synteny comparisons with other teleost species. The fact that many of these SNPs came from ESTs whose protein functions are known in other groups allows us to estimate the position of functionally important loci in *Lucania* (see Table S1). This is the first linkage map for any member of the Fundulidae family, a group that exhibits an extraordinary ability to tolerate and adapt to physiological extremes (Burnett *et al.* 2007).

By combining our linkage maps with actual chromosome counts, we found strong evidence for a major chromosomal rearrangement between *L. parva* and *L. goodei*. Our metaphase spread showed that a large metacentric chromosome was present in *L. parva* and absent in *L. goodei* (see arrow in Figure 2). Our linkage maps indicate that the largest *L. parva* LG (LG 1, 46.4 cM) is syntenic with two smaller *L. goodei* chromosomes (LG 1A and 1B). Comparisons of interspecific linkage maps have previously been used in other taxa to identify chromosomal rearrangements between species (Tanksley *et al.* 1992; Burke *et al.* 2004). Several pieces of evidence suggest that the metacentric chromosome is due to a Robertsonian fusion of two smaller acrocentric chromosomes in the *L. parva* lineage rather than a chromosomal fission event in the *L. goodei* lineage (a metacentric chromosome becoming two acrocentric chromosomes). First, *Fundulus parvipinnis* is the closest relative to *Lucania* (Whitehead 2010), and its karyotype is similar to *L. goodei* with 1N = 24 (Chen 1971). Second, *L. goodei* LG 1A is syntenic to chromosome 3 in medaka, and LG 1B is syntenic to chromosome 11. The fact that each of these two LGs map to a single LG in medaka suggests that they are unlikely to reflect the outcome of a fission event. Our evidence for a chromosomal fusion clarifies a previous report (made without any supporting data) that *L. parva*’s karyotype of 1N = 23 deviates from the typicial fundulid karyotype of 1N = 24 (Uyeno and Miller 1971). However, our evidence for a fusion should be further verified using *in situ* hybridization to determine that LG1 markers are indeed found on the metacentric chromosome in *L. parva*.

With the exception of the fused chromosome, comparisons between our maps reveal that large-scale structure is widely conserved between species. At the level of the linkage group, we found high preservation of synteny between *Lucania* linkage groups. We also found preservation of synteny between *Lucania* and medaka chromosomes. Each *Lucania* chromosome could be assigned unambiguously as syntenic to a single medaka chromosome. This result is consistent with findings from other teleost linkage maps and genomes (Schartl *et al.* 2013). This degree of synteny will facilitate future comparisons with other related teleost species for which genomic resources are emerging, such as guppies (Tripathi *et al.* 2009; Fraser *et al.* 2011) and fundulids (Cossins and Crawford 2005; Burnett *et al.* 2007). Currently, the fusion we document in *Lucania* (between chromosomes that are syntenic to medaka 3 and 11) is unique among related fish species for which linkage maps exist. Guppies (*Poecilia reticulata*) also possess a fused chromosome, but it has occurred between two chromosomes that are syntenic to medaka 2 and 21 (Tripathi *et al.* 2009). Tilapia (*Oreochromis niloticus*) possess two fused chromosomes: one between chromosomes that are syntenic to medaka 2 and 4, and another between chromosomes syntenic to medaka 6 and 12 (Liu *et al.* 2013).

Our comparisons of synteny at the marker order level revealed significant marker order preservation in only half of the linkage groups between *L. parva* and *L. goodei*. Our estimate of 50% collinear markers is much lower than a previous estimate of marker order preservation between hybridizing populations, which found that 83% of markers between incipient species of whitefish (*Coregonus clupeaformis*) were collinear (Rogers *et al.* 2007). Comparisons of chinook salmon and rainbow trout (genus *Oncorhynchus*) also suggest high preservation of marker order between congeners (Naish *et al.* 2013). Our study differs from these previous ones because we used SNPs derived from ESTs rather than microsatellites and consequently had a much denser distribution of markers on our map. However, further work is needed to determine if the low marker-order synteny in *Lucania* is genuine or simply the result of (1) low number of orthologous markers on some linkage groups, (2) genotyping errors, or (3) map construction errors due to merging maps from multiple families within each species. If genuine, then the low marker-order synteny found in *Lucania* would be indicative of small-scale rearrangements, which could potentially contribute to the reduction of gene flow and the evolution of reproductive isolation.

Our linkage maps for *Lucania goodei* and *L. parva* showed that a large-scale genomic rearrangement has occurred between species. This Robertsonian fusion may have aided divergence in these closely related species and helped to maintain species boundaries in zones of contact by suppressing recombination in hybrids. Other fundulid species also differ in karyotype, and our work may give insight into the mechanisms by which these changes occur. These maps will enable the use of numerous genomic techniques to determine how reproductive isolation has evolved in *Lucania*. The maps will also further a general understanding of the evolution of many other unique traits in this group including vision, color pattern, and salinity tolerance.

## Supplementary Material

Supporting Information
