# Supplementary material for: Insight Into Genomic Changes Accompanying Divergence: Genetic Linkage Maps and Synteny of Lucania goodei and L. parva Reveal a Robertsonian Fusion
Source: G3 (Bethesda). 2014 Jun 3;4(8):1363–72. doi: 10.1534/g3.114.012096 (PMC4132168; doi:10.1534/g3.114.012096)
Supplement: Supporting Information [file supp_4_8_1363__index.html]

Insight Into Genomic Changes Accompanying Divergence: Genetic Linkage Maps and Synteny of Lucania goodei and L. parva Reveal a Robertsonian Fusion — Supporting Information 

# Insight Into Genomic Changes Accompanying Divergence: Genetic Linkage Maps and Synteny of *Lucania goodei* and *L. parva* Reveal a Robertsonian Fusion

## Supporting Information for Berdan *et al.*, 2014

**Files in this Data Supplement:**

- Supporting Information - Figures S1-S2, File S1, and Table S1 (PDF, 404 KB)
- Figure S1 - Synteny between linkage groups 2-23 in *L. parva* and *L. goodei*. (PDF, 281 KB)
- Figure S2 - Summary of synteny comparisons between *Lucania* and medaka linkage groups. (PDF, 108 KB)
- File S1 - Supplemental Methods (PDF, 85 KB)
- Table S1 - Protein coding matches for linkage map markers and their location on the *L. goodei* and *L. parva* linkage groups. (.xlsx, 57 KB)
