# Supplementary material for: Insight Into Genomic Changes Accompanying Divergence: Genetic Linkage Maps and Synteny of Lucania goodei and L. parva Reveal a Robertsonian Fusion
Source: G3 (Bethesda). 2014 Jun 3;4(8):1363–72. doi: 10.1534/g3.114.012096 (PMC4132168; doi:10.1534/g3.114.012096)
Supplement: Supporting Information [file supp_g3.114.012096_FigureS1.pdf]

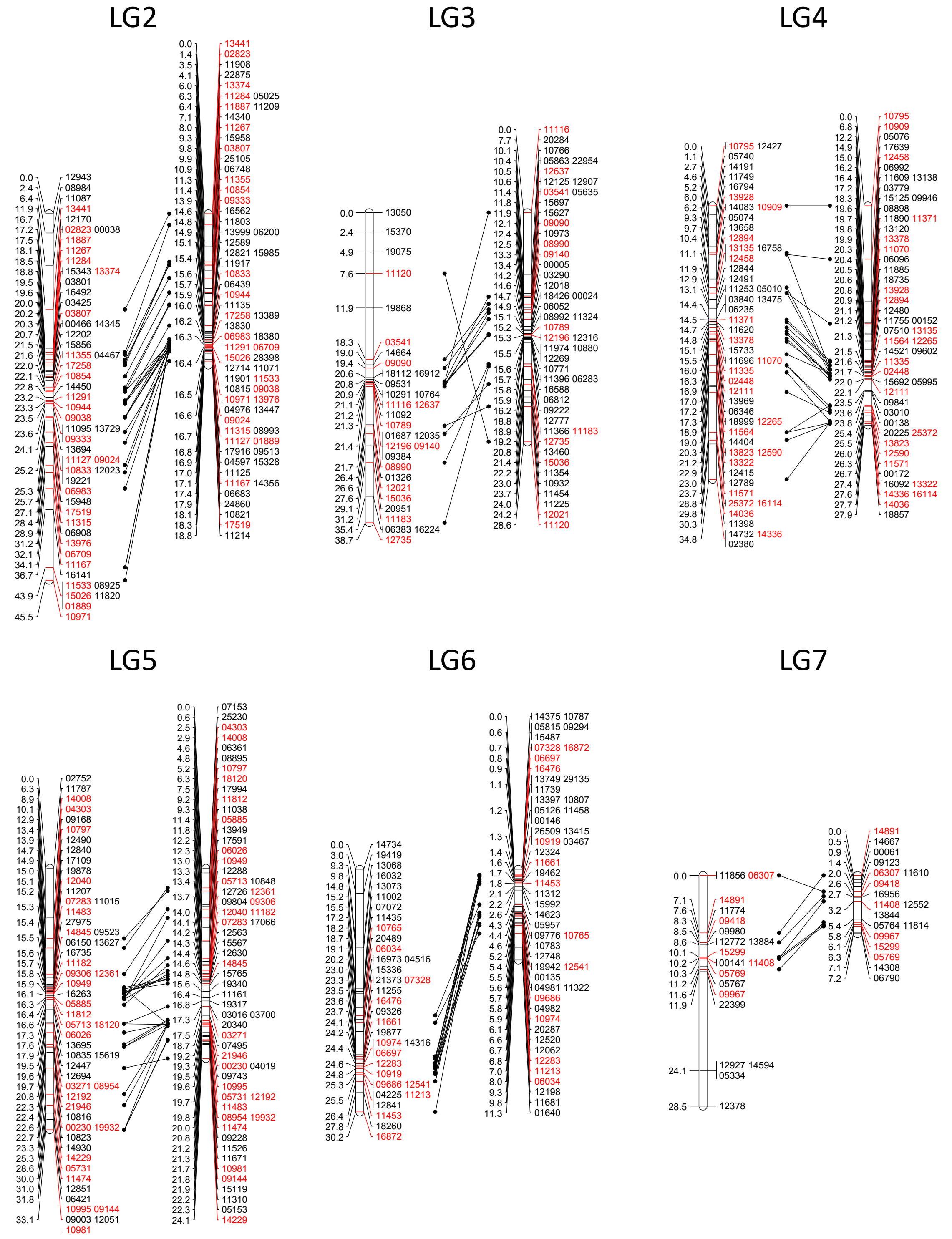
LG2
LG3
LG4
LG5
LG6
LG7

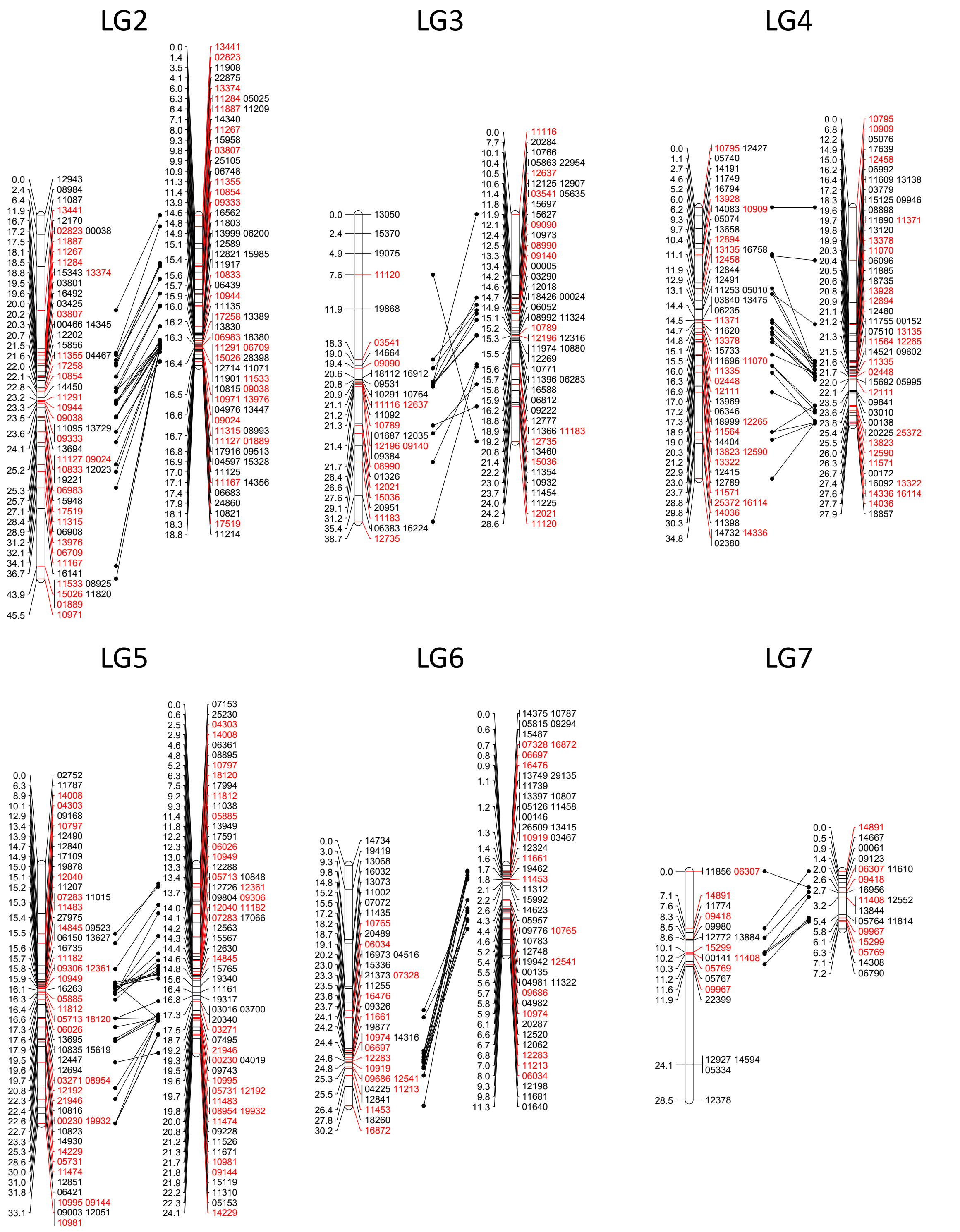
LG2
LG3
LG4
LG5
LG6
LG7

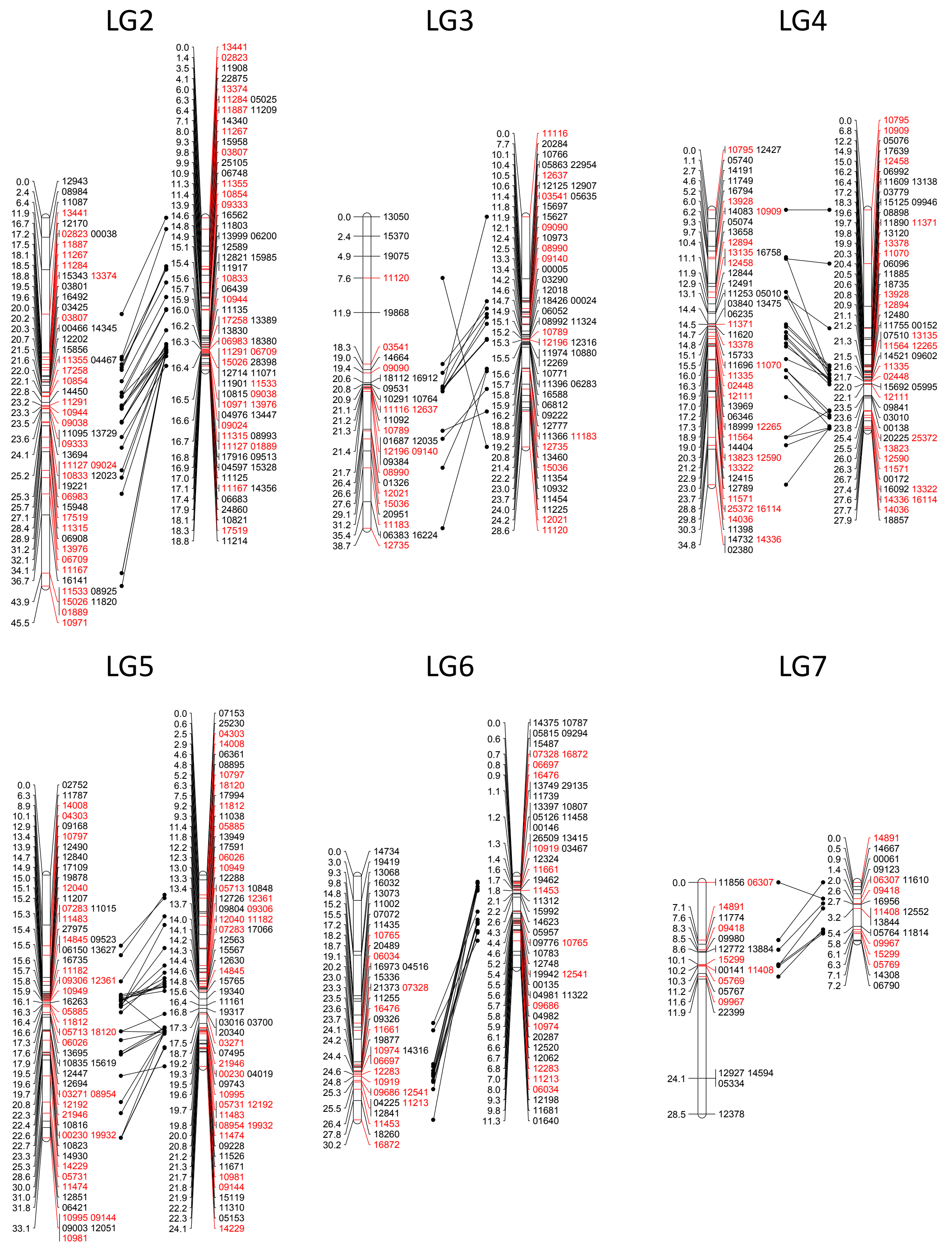
LG2
LG3
LG4
LG5
LG6
LG7

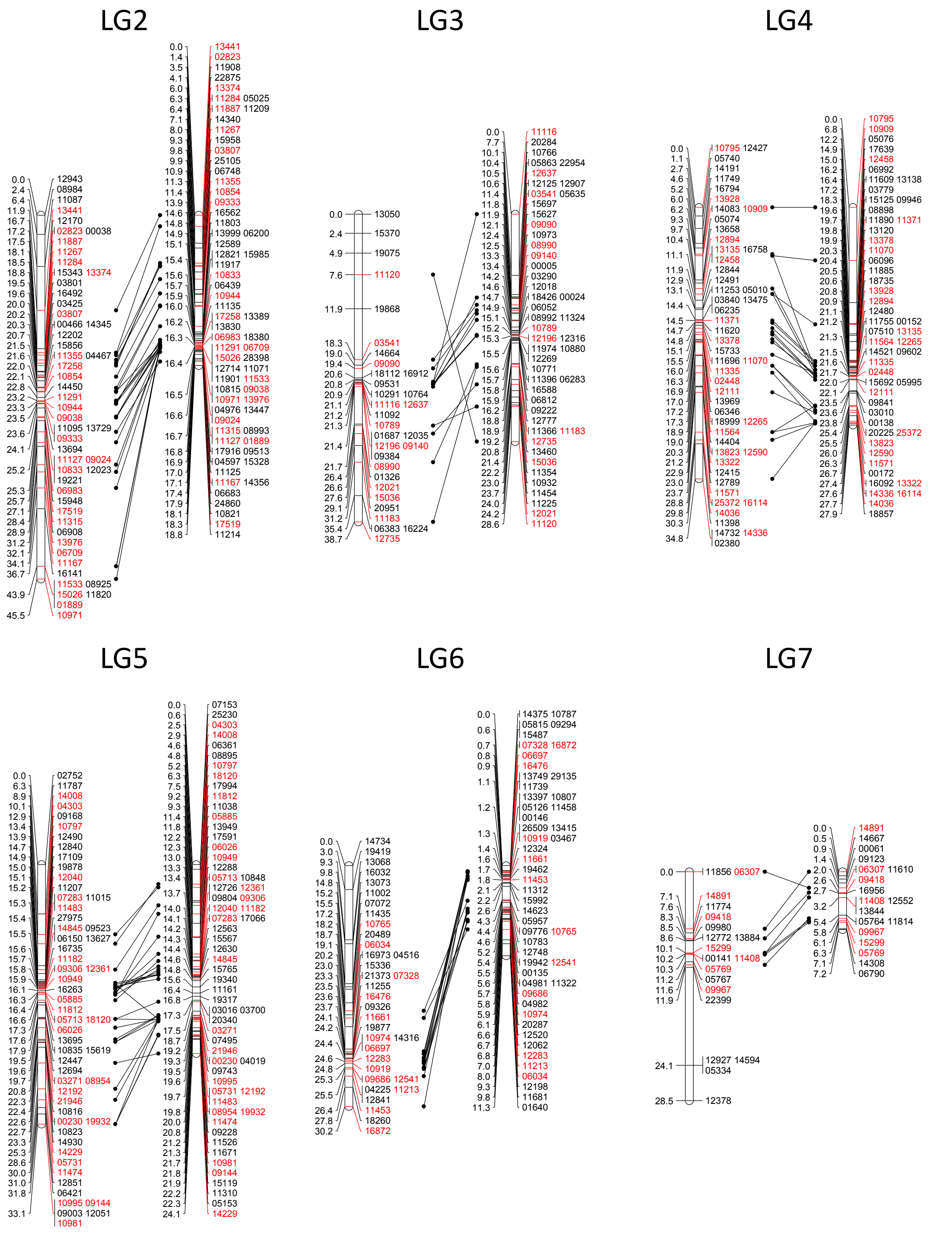

Figure S1 Synteny between linkage groups 2-23 in *L. parva* and *L. goodei*. For each linkage group, the *L. parva* group is on the left, *L. goodei* on the right. Orthologous SNPs between species are highlighted in red and connected by a line.
