# Supplementary material for: Insight Into Genomic Changes Accompanying Divergence: Genetic Linkage Maps and Synteny of Lucania goodei and L. parva Reveal a Robertsonian Fusion
Source: G3 (Bethesda). 2014 Jun 3;4(8):1363–72. doi: 10.1534/g3.114.012096 (PMC4132168; doi:10.1534/g3.114.012096)
Supplement: Supporting Information [file supp_g3.114.012096_FigureS2.pdf]

A.

	Medaka chromosome																							
	**3**	**11**	**9**	**5**	**8**	**16**	**4**	**20**	**17**	**15**	**12**	**21**	**7**	**6**	**24**	**14**	**10**	**13**	**19**	**23**	**18**	**2**	**22**	**1**
***L. parva* LG**																								
**1**	**29**	**13**																						
**2**			**32**								1													
**3**				**17**																				
**4**					**41**																			
**5**					1	**36**																		
**6**							**27**																	
**7**								**16**																
**8**									**16**															
**9**										**14**														
**10**											**21**													
**11**												**10**												
**12**				1									**30**											
**13**		1												**19**										
**14**															**34**									
**15**																**29**								
**16**											1						**29**							
**17**																		**15**						
**18**																			**31**					
**19**																				**17**				
**20**																					**12**			1
**21**																						**8**	6	
**22**																	1						**20**	
**23**																	1							**15**

B.

	Medaka chromosome																							
	**3**	**11**	**9**	**5**	**8**	**16**	**4**	**20**	**17**	**15**	**12**	**21**	**7**	**6**	**24**	**14**	**10**	**13**	**19**	**23**	**18**	**2**	**22**	**1**
***L. goodei* LG**																								
**1A**	**28**								1											1				
**1B**		**16**				1																		
**2**			**46**								2													
**3**				**26**					1				2											
**4**					**42**														1					
**5**						**36**																		
**6**							**32**		1															
**7**								**8**												1				
**8**							1		**33**															
**9**										**14**														
**10**											**20**													
**11**												**14**												
**12**								1					**43**						1					
**13**	1	1		1										**15**										
**14**	1														**23**									
**15**																**32**								
**16**															1		**19**	1						
**17**																		**17**						
**18**																			**25**					
**19**																				**19**				
**20**																					**12**			
**21**																						**2**		
**22**																	1						**20**	
**23**	1		1							1							2							**17**

Figure S2 Summary of synteny comparisons between *Lucania* and medaka linkage groups. Bolded numbers along the diagonal show the number of orthologous SNPs on the linkage groups. Numbers off the diagonal are non-syntenic markers. (a) synteny between *L. parva* and medaka, (b) synteny between *L. goodei* and medaka.
