# Supplementary material for: Insight Into Genomic Changes Accompanying Divergence: Genetic Linkage Maps and Synteny of Lucania goodei and L. parva Reveal a Robertsonian Fusion
Source: G3 (Bethesda). 2014 Jun 3;4(8):1363–72. doi: 10.1534/g3.114.012096 (PMC4132168; doi:10.1534/g3.114.012096)
Supplement: Supporting Information [file supp_g3.114.012096_FileS1.pdf]

File S1

Supplemental Methods

Karyotype

For each species, animals of both sexes from multiple populations were used. To stimulate mitosis, individuals were injected intraperitoneally with ~20 µl of a 2% phytohemagglutinin (Invitrogen, Carlsbad, CA) solution. After 24 hours, fish were injected intraperitoneally with 5 µl of a 1% colchicine solution. Two hours later, the animals were sacrificed using MS-222 (Argent Chemical Laboratories, Redmond, WA). The gills were removed and placed in chilled distilled water to allow the cells to swell. The cells were allowed to swell for 30 minutes and then were placed in a 3:1 methanol:glacial acetic acid mixture for fixation. The gills remained in fixative for 30 minutes with changes to fresh fixative every 10 minutes. Slides were cleared in methanol, dipped in 100% ethanol, and then swirled in chilled distilled water. The gills were then dabbed on the slide 8-16 times and discarded. Slides were dried and cured overnight. Slides were stained with 5% Giemsa solution (ph 6.8) (Electron Microscopy Sciences, Hatfield, PA) and dried again. Permount (Fisher Scientific, Pittsburgh, PA) was used to mount the slides that were then visualized using a compound microscope.

Creation of population-specific EST libraries

Tissue samples were taken from the gills (1-2 arches), dorsal fins, eyes, brain, and the gonads (ovaries or testes). Some of the *L. parva* tissues were preserved in -80°C RNAlater (Ambion, Austin, TX, USA) or 99% ethanol prior to RNA extraction. Tissues were ground and then RNA extracted from the tissues using β-mercaptoethanol and QiaShredder columns (Qiagen, Valencia CA) (Carelton 2011). RNA was purified using RNeasy mini kits (Qiagen, Valencia CA) and treated with Turbo DNase (Invitogen, Grand Island, NY).

Indexed RNA-seq libraries were constructed for each population with the TruSeq RNA Sample Preparation Kit (Illumina, San Diego, CA). Messenger RNA was selected from 1µg of high quality total RNA. First-strand cDNA was synthesized with a random hexamer and SuperScript II (Life Technologies, Grand Island, NY). Double stranded DNA was blunt-ended, 3'-end A-tailed and ligated to indexed adaptors. The adaptor-ligated double-stranded cDNA was amplified by PCR for 15 cycles. The libraries were size selected on a 2% Ex-Gel (250 bp to 500 bp). Nucleic acid concentrations from the size-selected libraries were quantified with Qubit (Life Technologies, Grand Island, NY), and the average fragment size was determined on an Agilent bioanalyzer DNA7500 DNA chip (Agilent Technologies,

Wilmington, DE). Libraries were diluted to 10nM and fragments were sequenced from both ends on an Illumina HiSeq2000 using the TruSeq SBS sequencing kit version 2 (Illumina, San Diego, CA).

DNA Extraction

DNA was extracted using a modified version of the PureGene (Gentra Systems, www.gentra.com) extraction protocol over four days (see Supplemental Methods). Tissue samples were placed in 600 µl of cell lysis solution (0.1 M Tris, 0.077 M EDTA, and 0.0035 M SDS) with 3 µl of Proteinase K (20 mg/ml) and incubated at 65°C overnight. Protein precipitation solution (200 µl; Qiagen, Valencia, CA) was added. The sample was stored at 4° C overnight and then centrifuged at 12.6 x 10^3 rpm for 5 minutes. The supernatant was transferred into 600 µl of isopropanol kept at -20°C overnight, then centrifuged at 12.6 x 10^3 rpm for 4 minutes to precipitate the DNA. The DNA was washed in 600 µl of 70% ethanol, centrifuged again. The supernatant was removed, and the DNA dried and then rehydrated in 30 µl of TE. Sample concentration and quality were verified using a Nanodrop spectrophotometer (Thermo Fisher Scientific, Waltham, MA).

SNP Selection

The alignments were exported to MAQ (Mapping and Assembling with Quality) software (see Fuller and Claricoates, 2011 for details) for SNP detection. Program parameters for calling SNPs were as follows: the minimum read depth to call a SNP was 50, and the maximum quality of reads covering a SNP was set at 50. Diagnostic SNPs for each population were identified using its population pair as a reference. For example, for *L. goodei*, the consensus sequences for the Upper Bridge population were used as a reference to identify SNPs in the Everglades population. Then, the consensus sequence from the Everglades population was used as a reference to identify SNPs in the Upper Bridge population. Likewise, two sets of SNP searches were performed for the two *L. parva* populations. SNPs were considered to be diagnostic when they were identified unambiguously for both populations. There were many more diagnostic SNPs between the two *L. parva* populations than there were between two *L. goodei* populations. In order to increase the number of SNPs for *L. goodei*, SNPs were used that were fixed in one population but were segregating in the alternate population.

Candidate SNPs were submitted to Illumina for initial evaluation for suitability for the Infinium Genotyping Assay. Candidate SNP analysis requires information on sequence orientation. Sequence orientation was determined by blasting contigs against amino acid sequences for all proteins in the NCBI database for medaka (*Oryzias latipes*), stickleback (*Gasterosteus aculeatus*), and mummichog (*Fundulus heteroclitus*). Illumina assigned each candidate SNP

a quality score that predicts its likelihood of success. All candidate SNPs with quality scores less than 0.6 were excluded. There were three classes of SNPs: *L. parva*-specific SNPs were SNPs that are segregating within *L. parva*, *L. goodei*-specific SNPs were SNPs that are segregating within *L. goodei*, and between species SNPs were SNPs that are fixed or almost fixed between the two species. The between species SNPs were designed for another study. SNPs were selected to (1) maximize quality score, (2) maximize the number of contigs that included *L. goodei*-specific*, L. parva*-specific*,* and between species SNPs, and (3) maximize the probability of being diagnostic for its given task. The custom Infinium bead chip held probes for 4,545 SNPs. Of these, 1,497 were candidate SNPs for *L. goodei*, 1,369 were candidate SNPs for *L. parva,* and 1,679 were candidate between species SNPs.

Flow cytometry

The genome size of both species was estimated using flow cytometry in order to determine how much recombination was occurring per megabase. Four *L. goodei* and four *L. parva* individuals were collected from the Lower Bridge site on the Wakulla River, Florida. The DNA content of erythrocyte nuclei was measured using flow cytometry. Blood samples were taken via cardiac puncture and were suspended in a solution of 250 mM sucrose, 40 mM trisodium citrate, and 5% dimethyl sulfoxide and stored at -80°C until further use. Blood was taken until the solution was light pink in hue. For each sample, 100 µL of the sample blood and 100 µL of a standard (*Betta splendens*) were pipetted into 20 mL of lysostaining solution (1g trisodium citrate, 1g Nonidet p40 substitute, 0.05g propidium iodide dissolved in 1L deionized water). For each blood sample, at least 10,000 nuclei with coefficients of variation less than 3% were examined on a Becton Dickinson FACScan flow cytometer. PE laser fluorescence values are known to be directly proportional to DNA content and the known DNA content of the standard can be used to estimate DNA content of the sample.

References

Fuller R. C., K. M. Claricoates, 2011 Rapid light-induced shifts in opsin expression: finding new opsins, discerning mechanisms of change, and implications for visual sensitivity. Mol Ecol 20: 3321-3335.
